# Are Sports Champions Also Anti-Epidemic Heroes? Quantitative Research on the Influence of Sports Champions’ Demonstration Effect on the COVID-19 Epidemic in China

**DOI:** 10.3390/ijerph19042438

**Published:** 2022-02-20

**Authors:** Junpei Huang, Shanlang Lin, Xiaoli Hu, Ruofei Lin

**Affiliations:** 1 School of Economics and Management, Tongji University, Shanghai 200092, China; 1830263@tongji.edu.cn (J.H.); 05069@tongji.edu.cn (S.L.); 2 International College of Football, Tongji University, Shanghai 200092, China; 21175@tongji.edu.cn

**Keywords:** COVID-19, demonstration effect, sports champion, physical exercise, mediating effect

## Abstract

What kind of role do sports champions play in the COVID-19 epidemic? Do they contribute to the mitigation of the epidemic by some pathway? In this paper, we empirically explore the influence and mechanism of the demonstration effect of sports champions upon the COVID-19 epidemic using COVID-19-related dataset of prefecture-level cities in China from 1 January 2020 to 17 March 2020. The two-way fixed effect model of econometrics is applied to estimate the result, the instrumental variable approach is adopted to address potential endogeneity issues, and socio-economic factors including public health measures, residents’ self-protection awareness, effective distance from Wuhan are also taken into consideration. The results show that the demonstration effect of champions in major sporting events increases the participation in physical exercise, which in turn reduces the possibility of being infected with the epidemic. An increase of one gold medal results in a 0.93% increase in the sports population, then leads to a 3.58% decrease in the cumulative case growth rate (*p* < 0.01). Further, we find that the effect is greater in regions with developed economies and abundant sports resources. Interestingly, it is greater in regions with less attention to sports, which again confirms the role of the demonstration effect.

## 1. Introduction

**Figure 1:**
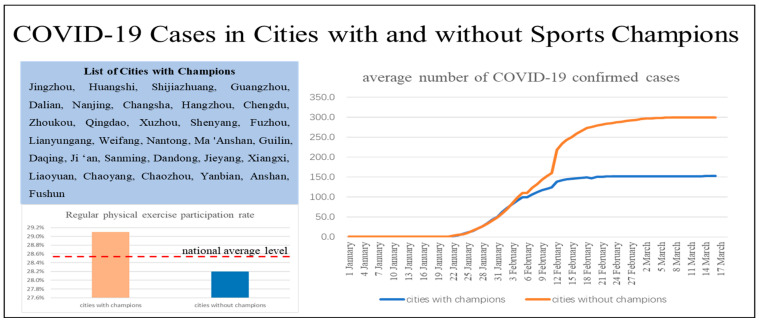
COVID-19 cases and sports population rate in Cities with and without sports champions.

Is there a correlation between the number of sports champions and the COVID-19 confirmed cases in a region? This is indeed an interesting question, and it seems that we can observe some evidence for it. Starting with a set of data in China, we counted the number of Chinese athletes who won gold medals at the 2016 Spring Olympics in Rio de Janeiro of prefecture-level cities according to the birthplace of the athletes, and it is obvious that there are fewer COVID-19 cases in regions with champions than those without champions (Figure 1). Moreover, it is revealed that except for Jingzhou and Huangshi, two cities in Hubei, the province with the first and worst outbreak in China, the number of confirmed COVID-19 cases in most champion cities is relatively small, even in provincial capitals with large and mobile populations, such as Fuzhou, Shenyang, Chengdu, Hangzhou, Changsha, Nanjing, and Guangzhou. At the same time, it can be observed that the share of the population that regularly participates in physical activity in champion cities is also higher than the national average and non-champion cities. Does it imply that there is indeed an association between the number of sports champions and the COVID-9 cases? If there is an association, what is the influencing mechanism? Athletic competition winners are often heroes in the eyes of people, especially young people, and are role models for them to learn from and imitate, so did these sports heroes who brought glory to the nation also make a great contribution to the epidemic prevention and control through some pathway?

Since the beginning of the pandemic, the correlation between physical exercise and the epidemic has been gradually attracting attention. It has been revealed that the morbidity and mortality of COVID-19 are correlated to excessive inflammation, failure in the adaptive immune response leading to an increased viral load [1], while doing physical exercise can not only improve cell-mediated and humoral immunity, promoting enhanced immunosurveillance [2,3], but also increase the antibody response of vaccination [4]. Immune aging is corelated to an increase in individuals’ susceptibility to infections, due to the decline in immune function, which can occur at any stage of the immune response. Such changes can be seen, especially when associated with emotional stress [5]. Therefore, a considerably higher mortality rate is observed in patients with advanced chronological age [6]. The precarious metabolic health is considered the main risk factor for the development of severe forms of COVID-19. This may occur in T2DM, obesity and MS, possibly due to immune dysfunction in synergism with pathophysiological complications of these comorbidities [7]. Regular physical activity (RPA) plays a positive role in immune aging and metabolic health [8]. In addition, physical exercise reduces the risk, duration and severity of viral infections [9]. Some focus on the fact that engaging physical exercise can enhance people’s immunity during the outbreak of the epidemic [10,11,12,13], and alleviate anxiety caused by public health measures such as quarantine and isolation [14,15], then reduces the rate of infection and death from COVID-19 [15,16,17]. Therefore, theoretically, reasonable physical exercise can strengthen the immune system and prevent the incidence of COVID-19 infections, the time of the infection, and mortality by COVID-19.

However, in recent years, the participation rate of physical exercise in China, especially teenagers, has shown a downward trend, and there is a similar situation in other countries. According to a survey of approximately 1.6 million students aged 11–17 years in 146 regions from 2001–2016, 81% of students did not achieve the level of “at least one hour of physical exercise per day” [18]. In particular, during the COVID-19 epidemic, people’s lifestyles and quality of life are greatly impacted [19], as a series of prevention and control measures negatively affect physical activity, with public stadium closures, home isolation, and social distance drastically reducing public physical exercise and online teaching reducing student physical activity [20,21,22,23], which adversely affects the physical activity of people, especially youth [24]. Obviously, actions are needed to motivate and increase participation in physical activity among the population, especially during the pandemic, more incentives should be given to youth to engage in physical activity to meet the WHO MVPA recommendations [24]. Sports events that attract public attention can effectively stimulate people’s passion for physical exercise, and the excellent performance of events or projects will promote participation in sports. There is evidence that the demonstration effect (also be defined as the trickle-down effect) of winners, especially champions in major sports events [25,26,27] can encourage people to become more active sports participants and expose residents to new sports activities [28,29].

Therefore, it seems intuitive that the demonstration effect of champions in major sports events can increase the population of engaging in physical exercise of a region, and then make an influence on the COVID-19 epidemic. Although substantial empirical evidence supports the effect of physical exercise on the epidemic, there is no existing literature linking COVID-19, physical exercise and the demonstration effect of major events or the winners. Our study is sought to fill this gap. The question we want to answer is: whether sports champion influences the COVID-19 epidemic, and what are the underlying mechanisms of the effect? To address the issue, in this study, we take the demonstration effect of sports champions, participation in physical exercise and COVID-19 epidemic into a unified research framework. Using COVID-19-related daily data of prefecture-level cities in mainland China, we explore the influence and mechanism of the demonstration effect of sports champions on the COVID-19 epidemic. The two-way fixed effect model of econometrics is applied to estimate the result, and we also take into consideration socio-economic variables including public health measures, residents’ self-protection awareness, effective distance to Wuhan.

The rest of the paper is structured as follows. Section 2 constructs the theoretical framework to explain the influencing mechanism. Section 3 specifies the materials and methods. Section 4 presents the results. Section 5 performs discussions. Section 6 draws conclusions and makes policy recommendations.

## 2. Theoretical Framework

The theoretical framework for this study is derived from the demonstration effect of sports championship and the immune function-improving effect of exercise, and we link the two theories into a unified analytical framework.

The demonstration effect has long been introduced into economic theory [29,30] and is now frequently used in the research in the field of sports [31]. It indicates that people are motivated by elite sports, sportspeople or sporting events so that they can participate in sports themselves [32]. Some empirical evidence also supports the demonstration effect of large sporting events and winners of them. Evaluations following the 1992 Summer Olympics, the 1994 World Cup and the 2002 Winter Olympics reports increased membership in sports clubs/organizations for both host and non-host area residents [27,31,33,34]. An annual survey conducted only 12 months after the 2000 Sydney Olympics shows increased participation rate in beach volleyball, water polo and track and field [35], sports in which Australian athletes performed well during competition. Potwarka and Leatherdale [28] use nationally representative data from the Canadian Community Health Survey to explore whether the 2010 Vancouver Winter Olympics are associated with rates of recreational sports and physical activity among youth living in the Olympic venue area. It is revealed that from 2007 to 2008 (pre-event) to 2009 to 2010 (one year before the event and event year), Richmond Oval in British Columbia hosted speed skating competitions during the games, a venue that witnessed a record number of Canadian women medalists in long track speed skating. They hypothesize that the spectacular performances at these arenas may be particularly inspiring to young women living in this particular region, especially those who are able to witness these medal performances live.

Some scholars have argued that demonstration effects are associated with hosting events [28], sporting success [36], and athlete role models [26], and this study is concerned with the demonstration effects generated by athlete role models. The concept of role model is extensively used in the field of economics. It is used when explaining the effect of important people on decisions making in general [37] and about career choice [38] especially entrepreneurship [39], and it is also explored the effect on students’ performance [40]. Moreover, it also plays an important role in learning [41], socialization [42], and the behavior of consumers [43]. Despite wide acceptance and applications, it lacks a clear definition of the concept [44]. It is popular among researchers and practitioners; however, an overuse due to lack of thought greatly diminishes its value. To overcome the problems on concept, Jung [45] and Gibson [46] provide a modern approach to role models, where it must satisfy two characteristics. The first is that it can be featured by distinguished achievement, and the second is to be considered similar in some respects by whom think the role models are worthy learning from. Factors such as outstanding achievement and similarity which are inherent in the concept of role models naturally also determine the choice of role models. For instance, Fleming et al. [47] finds that the choice of role models in British male youth rugby players is influenced by the technical competence, physical features, and temperament of professional players. Substantial empirical evidence shows that young people choose famous athletes as role models [47,48,49], in which boys are significantly more likely to select an athletic role model than girls [50], and sports viewers also choose professional athletes as role models [51]. In this study, the athlete who wins the championship is defined as a specific role model. Those role models who achieve remarkable achievements in sporting events can encourage non-participants to engage in sporting behavior or inspire those who have already played sports to do it more often.

Another part of the theoretical framework is the immune system improving effect of physical exercise, which helps to reduce the possibility of COVID-19 infection. Although it is still not quite clear in the understanding of the pathogenic mechanism of SARS-CoV-2 infection, there is an agreement in the existing scientific literature regarding the important role of the immune system in COVID-19 susceptibility, progression and outcome. It is revealed that the imbalance in innate and adaptive immune responses are featured mainly by changes such as cytokine storm and lymphopenia [52], in addition to the disorders in coagulation and host-related conditions [7] such as obesity, metabolic syndrome and aging (immunosenescence), is among the factors notoriously associated with a worse prognosis of infection [53]. While playing physical exercise performs the role of a regulator of the immune system. During and after physical exercise, pro- and anti-inflammatory cytokines is released, lymphocyte circulation increases, as well as cell recruitment [54,55]. As regard to the immune system in respiratory infections such as COVID-19, regular and at appropriate intensity levels of physical exercise helps to enhance immunovigilance and improv immune competence, which is beneficial to the control of pathogens, a fact that is considered more important regarding the immunosenescence and susceptibility of the elderly population to severe infection [56]. Other positive influence related to host factors, such as prevention or reduction of overweight, increased physical and cardiopulmonary conditioning, attenuation of the systemic pro-inflammatory and pro-thrombotic states, decrease in oxidative stress, improvements in glycemic, insulinic and lipidic metabolisms [3].

**Figure 2:**
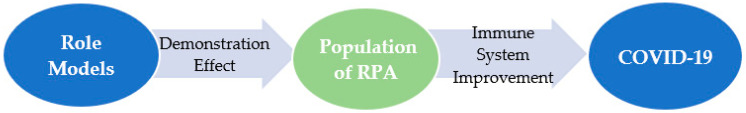
How do sports champions affect COVID-19.

In the theoretical framework, it is the number of regular physical activity (RPA) participants that links the demonstration effect of sports champions to the immune function improvement effect of physical activity. As mentioned above, the number of regular physical activity participants is influenced by the demonstration effect of sports champions, in which the demonstration effect of sports role models motivates the people to actively participate in physical activity. Then, the number of regular physical activity participants influences the number of COVID-19 infections, because the more people who participate in physical activity regularly, the more people who improve their immune function, the fewer people who are at risk of COVID-19 infection. The theoretical framework of this study is shown in Figure 2.

However, how much does the demonstration effect of sports champions work is related to external factors. External factors include many aspects, the most important of which are the level of economic development, sports facilities and media communication. Pawlowski et al. [57] find that households with higher incomes are more likely to spend money on sports. Wicker et al. [58] analyze 21 different sports and find that an additional hour of sports per week led to an annual sports-related expenditure by 263 euros. Some literature proves that sports facilities and their availability have a constant effect on sport participation [59,60]. Public knowledge and attention to sport are influenced by the information provided by the mass media [60,61,62,63]. External factors such as the level of economic development, sports facilities and media communication vary considerably across cities, so the size of the demonstration effect varies across cities, as does the number of people regularly participating in sport. Therefore, it is possible that there is a significant regional heterogeneity in the effect of demonstration effects generated by sports champions on COVID-19 epidemic.

Based on the analysis above, the following three hypotheses are proposed in this study:

1. The more sports champions there are, the fewer people are infected with COVID-19.
2. The demonstration effect of sports champions influences the COVID-19 epidemic by increasing the number of people who engage in physical exercise, i.e., the number of people who regularly participate in physical exercise is the mechanism by which champions influence the epidemic.
3. The effect of sports championships on the number of COVID-19 infections is heterogeneous depending on urban conditions.

## 3. Materials and Methods

### 3.1. Study Area

**Figure 3:**
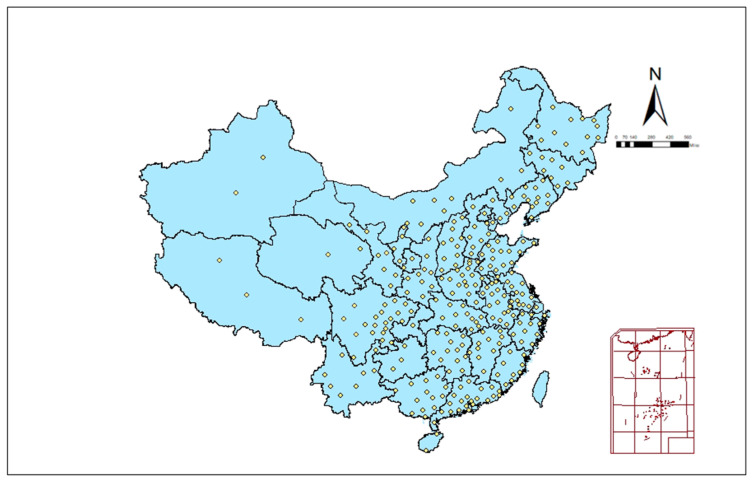
279 prefecture-level cities selected in this study.

Our study includes 279 prefecture-level administrative regions in China, covering all provinces in mainland China, and the vast majority of prefectural cities are in our sample (see Figure 3). The missing ones are mainly autonomous cities dominated by ethnic minorities in Tibet, Xinjiang, Inner Mongolia, and southwest China, which are excluded due to the high number of missing data and are not usable and representative. Our selected 279 urban cities cover more than 98% of China’s total population and more than 99% of GDP, and the sample is representative and widely used in studies of China [64,65,66].

### 3.2. Empirical Model

In this paper, econometrics approach is used to empirically explore the influence of the demonstration effect of major sports events on the COVID-19 epidemic. Econometric approach has been extensively applied to analyze the impact of a factor on economic growth. Similarly, COVID-19 infections typically increase exponentially with an alterable rate and can be influenced by other factors [67]. Therefore, it is appropriate to apply econometrics techniques to COVID-19 epidemic related research. In this paper, we use the two-way fixed effect model of econometrics to control both time-invariant individual heterogeneity and the individual-invariant time heterogeneity, the model is constructed as follows:

(1) $$lnrate_{i,t}=\alpha +\beta_{1}gold_{i}+\overset{n}{\underset{2}{\sum }}\beta_{k}X_{kit}+\theta t+\delta_{c}+\delta_{t}+\varepsilon_{i,t}$$

where, $rate_{it}$ is the is the actual COVID-19 cumulative confirmed case growth rate of city *i* in date *t*; $gold_{i}$ is the main explanatory variable, denoting the total number of gold medals in major sports competitions won by city i in 2019. $\beta_{1}$ is the coefficient of interest, which is the outcome we most care about. If $\beta_{1}<0$, then it indicates that the number of gold medals is negatively correlated to the growth rate of confirmed cases.$\;X_{k}$ is control variable, including public health measures (measure), residents’ awareness of protection (awareness), effective distance from Wuhan (distance), population density (popdens), traffic conditions (transport, passenger). $\delta_{i}$ is a region fixed effect,$\;\delta_{t}$ is a time fixed effect, and t is the time trend to control the variation trend of the explained variable over time.$\;\varepsilon_{it}$ is stochastic disturbance term, we estimate the standard deviation using cluster-robust standard error [68]. To address possible biased estimation results led by endogeneity issues, on the basis of baseline regression model, this paper selects the number of stadiums as the instrumental variable for explanatory variable (number of gold medals) and re-estimates the results using two-stage least squares method (2SLS) and limited information maximum likelihood method (LIML), respectively.

### 3.3. Variables

#### 3.3.1. Explained Variable: Actual Cumulative Confirmed Case Growth Rate

The explained variable in this paper is actual cumulative confirmed case growth rate:

(2) $$Actual\;cumulative\;case\;growth\;rate{\;}_{it}=(Actual\;case_{it}-Actual\;case_{it-1})/Actual\;case_{it-1}$$

This time period is used as the study sample because China achieved zero confirmed cases nationwide for the first time on 17 March 2020, and only a very few localities have experienced recurrent cases since then.

Earlier studies indicated that the mean incubation period of COVID-19 is 5.2 days [69], so we use the fifth forward term of reported cases as the proxy for actual cases:

(3) $$reported\;case_{it}=actual\;case_{it+5}$$

#### 3.3.2. Explanatory Variable: Number of Gold Medals in Major Sports Events

**Figure 4:**
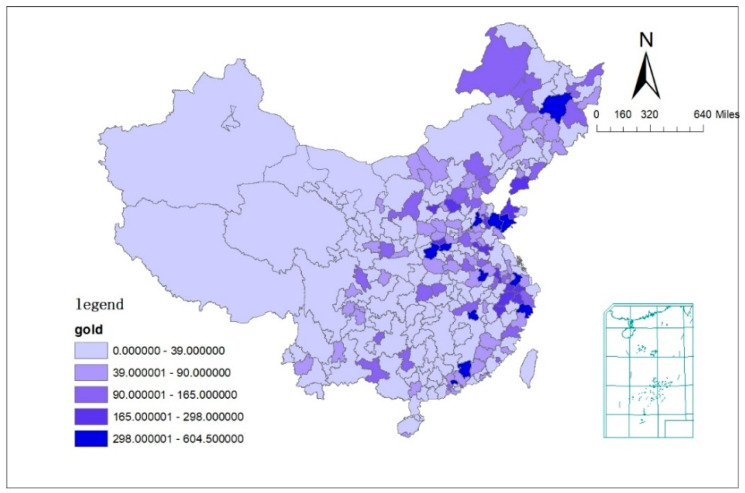
The number of gold medals in 2019 in China.

To capture the demonstration effect of sports champions, this paper selects the number of gold medals won in provincial-level and above sports events of each city in 2019 as a proxy variable for the explanatory variable. In this paper, provincial-level and above sporting events include international and intercontinental competitions (Olympic Games, Asian Games, World Championships, etc.), national competitions (National Games, National Winter Games, etc.), and provincial competitions (sporting events held by each province itself). When there is a case of sharing a gold medal with another province or city, this championship award is counted as 0.5 gold medals. The distribution of number of gold medals is shown in Figure 4. It can be seen that the cities with more gold medals are Qingdao, Jinan, Suzhou, Harbin and Hefei, with the numbers of 604.5, 565, 489, 445 and 392, respectively.

#### 3.3.3. Mediator (Mechanism) Variable: Sports Population

In this article, the increase in the number of people engaged in physical exercise due to the demonstration effect of sports champions is the influence mechanism by which sports champions affects the COVID-19 epidemic. Hosting international elite sporting events is a potential way to enhance sport participation as they can expose youth to new sporting opportunities and motivate them to become more active sport participants [28]. This phenomenon is known as the demonstration effect [25,26], where people are inspired by elite sports, sportspeople, or sporting events to involve themselves in physical activity [27]. Then, physical exercise not only improves cell-mediated and humoral immunity and promoting enhanced immunosurveillance [70], but also significantly increases antibody responses to vaccination [4], which in turn reduces the possibility of people infected by the COVID-19. Therefore, the number of people who regularly participate in physical exercise is the mechanism variable in this paper, i.e., the sports population. Considering the availability of data, we calculated the sports population in 2019 based on the total population and the proportion of the population regularly participating in physical exercise in 2019, as a proxy for the mediating variable.

(4) $${\mathrm{sports}\;\mathrm{population}\;}_{i}=total\;population_{i}\times {\mathrm{proportion}\;\mathrm{of}\;\mathrm{the}\;\mathrm{population}\;\mathrm{who}\;\mathrm{regularly}\;\mathrm{take}\;\mathrm{part}\;\mathrm{in}\;\mathrm{physical}\;\mathrm{exercise}}_{i}$$

#### 3.3.4. Control Variables

Public health interventions (measures). We refer to the method used in Lin et al. [71] to construct the scoring data for public health interventions. Comprehensive intervention efforts implemented in China has significantly mitigated the COVID-19 pandemic, particularly during the early phases of the outbreak. Therefore, it is necessary to incorporate the factor into the model. We carefully evaluate the prefectural score of public health interventions by manually collecting information or announcements released by the prevention and control headquarters of each prefecture-level cities.

Residents’ self-protection awareness (awareness). The public’s risk perception of the epidemic helps them to do timely personal protection, reduce unnecessary exposure, and thus decrease possibility of becoming infected [72]. In this paper, the Baidu search index of the term “mask” is used as a proxy variable to reflect the awareness of self-protection among residents.

Effective distance from Wuhan (distance). Studies indicated that effective distance rather than geographical distance is more important when predicting the spread of outbreaks [73]. So, we refer to the method in Lin et al. [74] to estimate the effective distance between each prefecture-level city and Wuhan, which reported the first and most COVID-19 cases in China.

Moreover, we control population density (popdens), total number of operating vehicles (transport), and passenger volume (passenger) to reflect flow of people and traffic in each city.

### 3.4. Data Source

The dataset used in this research covers 279 prefecture-level cities in mainland China, and the sample period is from 1 January 2020 to 17 March 2020. The number of cumulative confirmed cases is collected from the announcement of National Health and Health Commission. The number of sports gold medals is manually collated from the statistical yearbook, statistical bulletin and Sports Bureau of each prefecture-level city. The proportion of people who regularly participate in physical exercise used to calculate the sports population is compiled from official bulletins such as the National Fitness Report, National Fitness Development Survey Bulletin and National Fitness Action Plan issued by provinces and cities. Specific measures and timing of public health interventions come from official release from prevention and control headquarters of prefecture-level cities. The population migration data which is to calculate indicator of effective distance is available in Baidu Migration website (http://qianxi.baidu.com/ accessed on 24 November 2021). The attention of “mask” is obtained from Baidu Index website (https://index.baidu.com/v2/index.html#/ accessed on 24 November 2021). Variables of total population, population density, GDP per capita, total number of operating vehicles and passenger volume are available in China City Statistical Yearbook. The descriptive statistics of related variables are shown in Table 1.

## 4. Results

### 4.1. More Champions, Fewer Cases?

The baseline results are reported in Table 2 column (1). The explained variable is the actual cumulative cases growth rate (rate), and the explanatory variable is the number of gold medals (gold). It is shown that the coefficient of the number of gold medals is significantly negative, indicating that on average, there is a negative causal relationship between the number of champions and the confirmed case growth rate, that is, in cities with a higher number of sports champions, there are fewer COVID-19 confirmed cases.

Additionally, control variables are also interpreted. The coefficient of public health measures is shown to be significantly negative, indicating that public health measures are critical for slowing down the pandemic. The coefficient of population density is significantly positive, indicating that higher population density makes it more difficult to isolate human-to-human interaction, which has a negative impact on preventing the spread of epidemic. The coefficient of residents’ self-protection awareness is significantly negative, which means that the improvement of residents’ awareness of self-protection can effectively reduce the possibility of being infected. The coefficients of total number of operating vehicles and passenger volume are both significantly positive, indicating that dense traffic will amplify the probability of pandemic. The coefficient of effective distance is significantly negative. The closer the effective distance to Wuhan is, the more likely there is to outbreak pandemic, as theoretically predicted.

To address the endogenous issue caused by inevitable omitted variables, in this paper, we re-estimate the result apply the instrumental variable approach. Instrumental variable analysis is an established inference framework to investigate causal relationships from observational data in the presence of possible confounding, which has been widely applied in econometrics and epidemiology. The two criteria for selecting tool variables are: first, there must be a substantial association between the instrumental variables and the endogenous explanatory variables; second, the instrument variable must be exogenous. In this paper, the number of stadiums is selected as the instrument variable for the explanatory variable. The construction of stadiums provides residents with convenient sports venues for physical exercise, creates a good sports atmosphere, and promotes the development of local sports, so the number of stadiums has a certain correlation with the number of sport champions. Moreover, the number of stadiums does not directly affect the COVID-19 epidemic, so it also satisfies the exogeneity condition, which can be used as an instrumental variable for the number of gold medals. We adopt the two stage least squares method (2SLS) and limited information maximum likelihood method (LIML) to re-estimate the coefficient, and the results are reported in Table 2 column (2) and column (3), respectively. It is revealed that the sign and significance of the explanatory variable estimated by IV results are in accordance with the baseline regression, which confirms the fact that in cities with a higher number of sports champions, there are fewer COVID-19 confirmed cases.

### 4.2. Robustness

This paper uses the following five methods for robustness test. First, we convert the number of gold medals into 0–1 dummy variable of whether it is high number of gold medals (gold_dummy). If the number of gold medals in that place is higher than the average value, then the variable takes the value of 1, otherwise it takes 0. Second, given that the outbreak was originally occurred in Hubei province and then quickly spread across the province, also, Wuhan, one prefecture-level city in Hubei was also the first city to lockdown, we eliminate Hubei province from the sample and re-estimate the coefficient. Third, the assumption used in baseline regression is that there is a five-day incubation period of COVID-19. In robust checks, we re-assume the incubation period of four and six days and then re-estimate the result, respectively. Fourth, given that there were no large-scale outbreaks in many provinces in January, we exclude the sample in January and re-estimated the results. Fifth, to exclude the effect of extreme values on the results, we winsorize all variables to a 1% bilateral tailing. The results are reported in Appendix A Table A1. Table A1 column (1) reports the result of converting the explanatory variables to dummy variables, column (2) reports the result of subsample without Hubei province, and columns (3) and (4) report the estimation results of adjusting for the length of the incubation period, columns (5) report the result of subsample without January, columns (5) reports the result after winsorization. As can be observed, the sign and significance of the coefficients are in line with the baseline regression, which implies that the result remains robust after changing explanatory variable, sample selection, and fundamental assumptions, indicating H1 is verified.

### 4.3. Does the Demonstration Effect Matter?

In this article, the increase in sports population induced by the demonstration effect of sports champions is the influencing mechanism by which the number of sports champions affects the COVID-19 epidemic. In order to further explore the mediating effect of the increase in sports population induced by the demonstration effect of sports champions, we refer to the method of Baron and Kenny [75] to perform the mechanism test. The steps are as follows:

(1) To test whether the number of sports gold medals significantly affects the sports population, that is, to test the demonstration effect of sports champions:

(5) $$lnsport_{i}=\alpha +\beta_{1}gold_{i}+\gamma X_{i,t}+\theta t+\delta_{c}+\delta_{t}+\varepsilon_{i,t}$$

(2) To test whether the increased participation in physical activity significantly reduced the COVID-19 confirmed case growth rate:

(6) $$lnrate_{it}=\alpha +\beta_{1}lnsport_{i}+\gamma X_{i,t}+\theta t+\delta_{c}+\delta_{t}+\varepsilon_{i,t}$$

(3) Introduce both the number of gold medals and sports population into the model:

(7) $$lnrate_{it}=\alpha +\beta_{1}gold_{i}+\beta_{2}lnsport_{i}+\gamma X_{i,t}+\theta t+\delta_{c}+\delta_{t}+\varepsilon_{i,t}$$

Table 3 reports the results of mediating effect test. Column (1) presents the result of Equation (5), it shows that the coefficient of the number of gold medals is significantly positive, indicating that the increase of the number of sports champions can improve the participation in physical activity, which verifies the demonstration effect of sports champions. Column (2) reports the result of equation (6), it can be seen that the coefficient of sports population is significantly negative, indicating that an increase in the number of physically active people could reduce the epidemic. Column (2) shows the result of equation (7), that is, the explanatory variable (gold) is also introduced on the basis of column (2). It can be seen that the coefficient of the sports population in column (3) is still significantly negative, but the magnitude of the coefficient decreases compared to model (1). It indicates that the sports population is the mediator variable by which the number of champions affect the number of confirmed cases, indicating H2 is verified.

### 4.4. Different Effect in Different Regions

We have verified that the demonstration effect generated by sports champions significantly increases the number of people participating in physical activity and thus reduces the number of COVID-19 cases. However, the differences in sports habits of people in different regions of China are not only affected by the development of the sports industry, but also closely related to the local resource endowment.

In order to further explore the regional heterogeneity of the effect, we divide the sample into high and low socioeconomic groups based on economic development (GDP per capita), sports resources (number of stadiums), and attention to sports (sports lottery sales), and then perform the subsample regression. The subsample regression results are reported in Table 4. Columns (1) and (2) present the results of the subsample regression by GDP per capita, and it can be seen that the effect is greater in high-GDP regions. Columns (3) and (4) report the results of the subsample regression by sports resources, which shows the influence of sports champions on the epidemic is greater in regions with sufficient sports resources. Columns (5) and (6) show the results of the subsample regression by attention to sports, and it is revealed that the effect is more significant in places with less attention to sports, then H3 is verified.

## 5. Discussion

This article explores the influence of sports champions on the COVID-19 epidemic. It is found that cities with a higher number of champions in major sporting event have a lower confirmed COVID-19 cases growth rate. The champions of major sporting events, as role models, have a demonstration effect on the public, which increases the number of regular physical activity participants, strengthens immune alertness and improves immune competence, and then in turn exerts a suppressive effect on the outbreak of COVID-19 infection. It has been well documented that physical activity reduces the possibility of COCID-19 infection [10,12,13,14,16,17], and there is already some literature on the demonstration effects of sport [26,27,28,75], especially the influence of sports stars on young people [61,62,76,77,78]. However, in the existing literature discussing the impact of physical activity on COVID-19 epidemics, there is no focus on the demonstration effect of sporting event champions [25,26,27,28,75] on the epidemic. We include the demonstration effect of sports champions, physical exercise, and COVID-19 epidemic in a unified research framework for the first time, which provide a new perspective on the impact of physical activity on the COVID-19.

The findings of this study also provide a new way of thinking for the field of sports health and sports management. The demonstration effect of sports champions as role models is “externality” in the economic sense. Externality, also known as the spillover effect, refers to the effect of a person or a group whose actions benefit or harm another person or group of persons without corresponding payment or compensation. It is an issue that needs to be faced by the government, communities, schools and media that how to better utilize the spillover effect of sports role models to drive the public’s enthusiasm for physical activity, especially to cultivate young people’s love and habit of sports. When organizing large-scale sports events, governments and sports management agencies should not only consider the spillover effect of sports in the accounting of investment and revenue, but more importantly, how to establish an interactive relationship between sports role models, youth, public physical activities, and public health, so as to give greater play to the demonstration effect of sports events.

Most of the research on the effect of physical exercise on COVID-19 epidemic is qualitative [10,79], or conduct experiments using individual samples [15] or questionnaires [80]. Moreover, quantitative analysis primarily relies on statistical approaches such as Pearson’s correlation. What approach to take to better analyze the causal relationship between physical activity and health has been a challenge in research on sports and health. To perform a more rigorous statistical analysis, in this study, we quantitatively explored the influence and mechanism of the demonstration effect of sports champions on the COVID-19 epidemic using prefecture-level statistics in China and applying a two-way fixed effects model of econometrics. Moreover, we take the number of stadiums as an instrumental variable for the main explanatory variables to address the potential biased estimates due to endogeneity issue.

The transmission of COVID-19 is complex and it is important to control as much as possible for other potential confounders to more effectively analyze the role of sports champions on the COVID-19. Concerning the spread characteristics of COVID-19, control variables such as human behavior patterns and economic and social conditions are taken into consideration. With regard to human behavior patterns, which are related to both population aggregation and accessibility and mobility, we calculate the effective distance proposed by Brockmann and Helbing [73] and include it in this paper. To date, the role of socioeconomic conditions on the spread of COVID-19 is unclear. Role of sports champions COVID-19 epidemic can be more accurately estimated by controlling key variables in the regression model that have been neglected in previous literature, including population density, residents’ self-protection awareness, economic development level, and health care conditions.

Compared to the existing literature, this paper focuses more on the effect of the Chinese government’s efforts on the epidemic. Different from other countries, China has taken unprecedentedly comprehensive and stringent measures during the COVID-19 outbreak. Therefore, the role of public health measures should not be ignored in the quantitative analysis of COVID-19 in China, yet most of the existing literature do not pay attention to this important factor. In this paper, based on Lin et al. [71], we further compile and evaluate a series of public health interventions implemented by prefecture-cities of China and take it into the empirical model including but not limited to school closure, travel restrictions, community control, social distancing, quarantine, isolation and tracking close contacts.

The aim of this paper is to investigate the mechanisms by which sports champions influence COVID-19 epidemic, and we suggest that an increase in the number of people participating in regular physical activity induced by the demonstration effect of champions as the mechanism. However, there are many factors that influence the level of physical activity, the most important of which includes socio-economic conditions [58], sports facilities [59] and media communication [61]. Pawlowski and Breuer [81] find that households with higher incomes are more likely to spend money on sports. Wicker et al. [58] analyzes 21 different sports and find that an extra hour of sports per week increases annual sports-related expenditures by 263 euros. Some literature proves that sports facilities and their availability have a significant impact on sport participation [59,60]. Moreover, public knowledge and attention to sport is influenced by the information provided by the mass media [61,62,63]. External factors such as the level of economic development, sports facilities and media communication vary widely in different cities, so there is regional heterogeneity of the effect.

Although there have been some studies exploring the effect of physical activity on COVID-19 in China [80,81,82,83], the majority of the research on the effect of physical activity on the pandemic in China relies on time series data with single samples, such as the whole nation or a specific city, or on cross-sectional data, such as questionnaires. Limited by sample size and data structure, these studies are unable to investigate the regional heterogeneity of the effect in more detail. However, there are huge differences in social and economic conditions among different regions in China, as well as great differences in people’s sports habits, which are not only affected by the development of the sports industry, but also closely related to local resource endowment. Therefore, we further perform heterogeneity analysis to explore the heterogeneous influence of sports champions’ demonstration effect on COVID-19 epidemic in regions with varying levels of economic development, sports resources and attention to sports, which supplements this issue. We find that the effect is greater in areas with higher levels of economic development. It is believed that there are better living conditions in economically developed areas, so residents have more energy to pay attention to sports events and they also care about their own health. Therefore, the emergence of sports champions in economically developed areas can drive more residents to engage in physical exercise, and thus reduce the probability of infection more effectively. Moreover, sports champions in regions with abundant sports resources have a greater impact on the epidemic. Adequate sports resources such as sports facilities can meet the growing demand for physical exercise of residents, avoid being unable to engage in physical activity due to problems such as lack of sports field, thus better playing the demonstration effect of champions. Interestingly, the effect is stronger in places where sports are less popular. In regions with low sports attention, the participation rate of national fitness will also not be very high, then the champions of major sports events, especially events attracting international attention such as the Olympic Games, will make a greater marginal effect, which further confirms the existence of the demonstration effect of sports champions.

As with all studies that attempt to find a causal relationship between physical activity and health issues, this study is confronted with the various complexities of sports role models, physical exercise participation, and the risk of COVID-19 infection. Since sport is a social activity determined by a variety of factors, it is difficult to quantify the demonstration effect of role models in isolation. Risk of COVID-19 infection is influenced by the host and environment, and it is also challenging to analyze how much of a role physical activity plays in the risk of COVID-19 infection. To establish a causal relationship among the three and to address the complexity of this topic, this study adopts an appropriate empirical methodology, in which we use a prefecture-daily panel dataset, and applies instrumental variables approach and multiple robustness tests, and control for other influences in various ways, especially considering the severe prevention and control measures in China. However, the question of causality is difficult to be adequately addressed.

One limitation of this study is that the data set does not include personal information such as differences in age, gender and culture. In fact, these factors may affect the demonstration effect of role models. For example, studies of Vescio et al. [84], Young et al. [85] demonstrates that factors such as gender and age affect the choice of role models, and also that those affect the physical activity level of young people [86,87]. In future studies, we will be committed to adopt more detailed survey methods and carry out questionnaire surveys under the legal framework of privacy protection and on the premise of obtaining consent of patients, so as to draw more accurate conclusions.

## 6. Conclusions

In this paper, we establish a dataset including COVID-19 cases, number of championships in major sports event, and the number of people who regularly take part in physical exercise, construct a theoretical framework, and empirically test the three hypotheses applying two-way fixed effect model of econometrics. The results show that the demonstration effect of champions in major sporting events increases the participation in physical exercise, which in turn reduces the possibility of being infected with the epidemic. In addition, the effect is regionally heterogeneous.

To the best of our knowledge, this is the first study that take the demonstration effect of sports champions, population of engaging physical exercise and the COVID-19 epidemic into a unified research framework. Moreover, we apply the two-way fixed-effect model utilizing daily panel data from prefecture-level cities in China, and also consider factors such as human behavior patterns, public health measures, and socio-economic conditions, drawing conclusions which is consistent with the intuition.

Our findings have several important policy implications. In the context of the COVID-19 pandemic, mitigation measures are an important strategy to reduce the risk associated with COVID-19 infection, including the use of personal protective equipment (PPE), compliance with hygiene procedures and social distancing measures, and actions to lead a healthier lifestyle, minimize stressors, and strengthen the immune system, such as regular physical activity. However, maintaining appropriate levels of physical activity during the lockdown, quarantine, isolation, and social distancing appears to be a challenge. Therefore, it requires local governments and communities to balance prevention and control measures with the opening of public sports facilities, and also requires schools to implement more diverse youth sports and advocate for students to maintain regular moderate physical activity during the outbreak while at home. In addition, the public should be advised to look for viable alternatives and choose new forms of physical exercise that suit their habits during the epidemic.

## Figures and Tables

**Table 1:** Descriptive statistics of variables.

Type	Variables	Description	N	Mean	sd	Min	Max
explained variable	rate	actual cumulative confirmed case growth rate	19,989	0.0581	0.4486	0	29
explanatory variable	gold	number of sports gold medals	19,989	2.7952	1.9651	0	6.4061
mediating (mechanism) variable	sport	sports population	19,989	3.4892	0.8301	1.5586	6.8084
instrumental variable	stadium	number of stadiums	19,989	2.7574	1.2301	0	5.8406
control variable	measure	score of public health intervention	19,989	5.2922	3.9633	0	10
	popdens	population density	19,989	4.7855	0.7808	2.8332	7.8047
	awareness	Baidu search index for “mask”	19,989	4.6223	2.1418	0	8.9115
	distance	effective distance from Wuhan	19,989	5.7160	1.8739	0	7.7846
	transport	total number of operating vehicles	19,989	6.7557	1.0996	4.1431	10.4860
	passenger	passenger volume	19,989	9.1692	1.2307	5.2575	12.7237

**Table 2:** Baseline Regression: OLS, 2SLS and LIML.

	(1)	(2)	(3)
		IV Results	
	OLS	2SLS	LIML
	Rate	Rate	Rate
gold	−0.0358 ***	−0.5302 ***	−0.7572 ***
	(0.0032)	(0.0481)	(0.0899)
measure	−0.3339 ***	−0.2593 ***	−0.5483 ***
	(0.0201)	(0.0307)	(0.0348)
popdens	0.2309 ***	0.1254 ***	0.1128 ***
	(0.0140)	(0.0231)	(0.0277)
awareness	−0.1366 ***	−0.1153 ***	−0.0869 ***
	(0.0058)	(0.0088)	(0.0145)
transport	0.1652 ***	0.4025 ***	0.7607 ***
	(0.0144)	(0.0313)	(0.0791)
passenger	0.0614 ***	0.1711 ***	−0.1015 ***
	(0.0106)	(0.0190)	(0.0211)
distance	−0.2433 ***	−0.1951 ***	−0.3093 ***
	(0.0052)	(0.0090)	(0.0154)
R-squared	0.835	0.635	0.265
Observations	19,989	19,989	19,989

All regressions include time trend, province fixed effects, time fixed effects, and control variables. Standard errors in parentheses, *** *p* < 0.01.

**Table 3:** Mediating effect test.

	(1)	(2)	(3)
	Sport	Rate	Rate
gold	0.0093 ***		−0.0330 ***
	(0.0004)		(0.0032)
sport		−0.3889 ***	−0.3077 ***
		(0.0519)	(0.0524)
R-squared	0.985	0.834	0.835
Observations	19,989	19,989	19,989

All regressions include time trend, province fixed effects, time fixed effects, and control variables. Standard errors in parentheses, *** *p* < 0.01.

**Table 4:** Heterogeneity exploration.

	High GDP	Low GDP	High Resources	Low Resources	High Attention	Low Attention
	Rate	Rate	Rate	Rate	Rate	Rate
gold	−0.0615 ***	−0.0175 ***	−0.0380 ***	−0.0349 ***	−0.0322 ***	−0.0423 ***
	(0.0045)	(0.0048)	(0.0050)	(0.0049)	(0.0037)	(0.0073)
R-squared	0.865	0.810	0.854	0.821	0.839	0.833
Observations	10,008	9981	9333	8856	14,878	5111

All regressions include time trend, province fixed effects, time fixed effects, and control variables. Standard errors in parentheses, *** *p* < 0.01.

## Data Availability

Data available on request due to restrictions, e.g., privacy or ethical.

## Appendix A

**Table A1:** Robustness.

	(1)	(2)	(3)	(4)	(5)	(6)
	Gold_Dummy	Exclude Hubei Province	Incubation Period = 4	Incubation Period = 6	Exclude January	1% Winsorization
VARIABLES	Rate	Rate	Rate	Rate	Rate	Rate
gold_dummy	−0.1555 ***					
	(0.0124)					
gold		−0.0430 ***	−0.0354 ***	−0.0363 ***	−0.0231 ***	−0.0356 ***
		(0.0029)	(0.0032)	(0.0032)	(0.0045)	(0.0031)
R-squared	0.835	0.833	0.835	0.834	0.745	0.837
Observations	19,989	19,125	20,267	19,711	8617	19,989

All regressions include time trend, province fixed effects, time fixed effects, and control variables. Standard errors in parentheses, *** *p* < 0.01.
